# Macropinosome quantitation assay

**DOI:** 10.1016/j.mex.2014.05.002

**Published:** 2014-06-02

**Authors:** Jack T.H. Wang, Rohan D. Teasdale, David Liebl

**Affiliations:** a Institute for Molecular Bioscience, The University of Queensland, St. Lucia, Brisbane 4072, Queensland, Australia; b School of Chemistry and Molecular Biosciences, The University of Queensland, Brisbane 4072, Australia; c Dept. Bacterial Pathogenesis and Cellular Responses, Institute of Sciences Research and Technologies/CEA-Grenoble, Grenoble, France

**Keywords:** Macropinocytosis, Macropinosome, Fluid-phase endocytosis, Dextran, Fluorescence microscopy, Quantitation of macropinocytosis, Amiloride

## Abstract

In contrast to phagocytosis, macropinocytosis is not directly initiated by interactions between cell surface receptors and cargo ligands, but is a result of constitutive membrane ruffling driven by dynamic remodelling of cortical actin cytoskeleton in response to stimulation of growth factor receptors. Wang et al. (2010) [13] developed a reliable assay that allows quantitative assessment of the efficiency and kinetics of macropinosome biogenesis and/or maturation in cells where the function of a targeted protein has been perturbed by pharmacological inhibitors or by knock-down or knock-out approaches. In this manuscript we describe a modified quantitative protocol to measure the rate and volume of fluid phase uptake in adherent cells. This assay:

• uses fluorescent dextran, microscopy and semi-automated image analysis;
• allows quantitation of macropinosomes within large numbers of individual cells;
• can be applied also to non-homogenous cell populations including transiently transfected cell monolayers. We present the background necessary to consider when customising this protocol for application to new cell types or experimental variations.

## Graphical abstract

**Figure:**
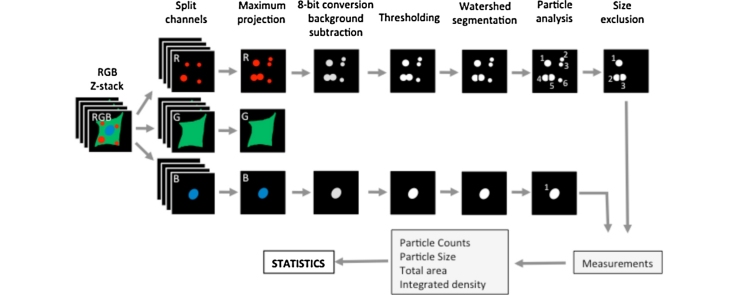

## Development of macropinosome quantitation **assay**

### Introduction

Although initially distinguishable by their large size – relative to other endocytic organelles – macropinosomes undergo homotypic fusion and fission [1] and may also converge with other endocytic pathways becoming visually intractable within minutes post internalization. In an attempt to quantify the rate of fluid uptake into macropinosomes several studies introduced horseradish peroxidase (HRP) [2,3] or Lucifer Yellow [4,5] as fluid-phase markers. In these assays the enzyme activity or the fluorescence associated with the marker was measured in the lysate of the cells via spectrophotometry. Such methods however, often neglect the substantial contribution of other endocytic pathways to the total amount of fluid-phase marker internalized by the cells, which in turn compromises the specificity of the assay. Since the rate of macropinosome formation, macropinosome numbers and their size are all characteristics that are suitable for investigation via microscopy, we focussed on development of a protocol for quantitative analysis of macropinocytosis using fluorescence imaging and semi-automated image analysis.

### Model cell line and timing of the fluid-phase uptake

Macropinocytosis has been studied extensively in antigen-presenting cells such as macrophages and dendritic cells, but many other cell types including epithelial cells and fibroblasts also exhibit macropinocytosis. With the intention to establish a model system which can be used as a platform to investigate the effect of candidate proteins or compounds on macropinocytosis in mammalian epithelial cells of non-cancer origin, we developed a quantitative image-based assay to analyze macropinocytosis in Human Embryonic Kidney cells (HEK293). We have previously shown that within the first 3–5 min post formation at the cell periphery, 80–90% of macropinosomes in HEK293 cells are positive for Rab5 and SNX5 but only 5% are positive for Rab7 suggesting that fusion with late endosomes and acquisition of endolysosomal markers begins from about 5 min post macropinosome formation [6]. Therefore, macropinosomes in HEK293 cells should be examined within this relatively short period to exclude those that may have converged with endolysosomal compartments. In the protocol we describe, cells are pulsed with a fluorescent fluid-phase marker only for 5 min and after a brief wash to remove non-internalized dextran and reduce the extracellular fluorescence, the cells are fixed to ensure that only early macropinosomes will be detected in the cells. Optionally, to further prevent macropinocytic uptake during the washing step, cells can be washed in cold PBS before fixation as macropinocytosis has been shown to be inhibited at 16 °C [7].

### Selection of a fluid-phase marker

One of the most convenient fluid-phase markers to study endocytosis by pulse-chase experiments is dextran, which has been also used as a fluid-phase marker for macropinosomes [8]. Dextran is a hydrophilic, non-digestible carbohydrate commercially available in a wide range of molecular sizes and photostable fluorophore conjugates with well differentiated excitation/emission spectra suitable for fluorescence microscopy. Our assay has been designed to quantify macropinosomes in HEK293 cells expressing GFP either as a reporter of shRNA-mediated knock-down or, as a tag of a protein of interest. For straightforward emission spectra separation we therefore use 10,000 MW dextran conjugated to tetramethylrhodamine (dextran–TMR) although dextran conjugated with any other suitable fluorophore can be used. Fluorescently conjugated dextrans are supplied as fractions of specific molecular weight commonly from 10 to 2000 kDa which corresponds to molecular dimensions (Stokes's radius) from 2.4 to 27 nm [9]. Using dextran of different molecular weight than 10 kDa should not significantly affect the kinetics of dextran internalization in larger endocytic structures but dextran solubility in aqueous buffers decreases with molecular weight what should be considered when preparing concentrated stock solutions.

### Size exclusion of endosomes

Another selective factor essential to maintain specificity of the assay is the size-exclusion of endosomal structures that do not meet the minimum limit for being classified as macropinosomes (0.2–5 μm in diameter) [10,11]. After 5 min of dextran uptake in HEK293 cells, dextran-positive structures that are both below and over this size threshold can be detected. At later time points (10 min), the number of large macropinosomes increases to some extent, however, a significant increase can be also observed in the number of endocytic structures that are smaller than 0.5 μm in diameter. This implies that a prolonged dextran pulse increases the likelihood of detecting false positives during quantitation of macropinosomes in the cells. Therefore only a short (5 min) pulse is recommended in the protocol of our assay which should minimize accumulation of dextran-positive endosomes internalized for example by clathrin-mediated pathway and measure up to 100–200 nm in diameter [12].

### Evaluation of semi-automated protocol

Each step of our assay [13] was systematically tested and optimized to maximize its sensitivity and efficiency. The semi-automated quantitation (see Graphical abstract) was assessed by applying the original protocol to a test image set of dextran-pulsed HEK293 cells and compared with results acquired by expert manual annotation. A minimal difference (13%) was found between numbers of macropinosomes identified and scored manually and those calculated by semi-automated image analysis protocol. This difference in accuracy was attributed to the limited ability of the semi-automated protocol to segregate adjacent macropinosomes and therefore an additional step was implemented in the assay which exerts watershed filtering and improves detection of individual macropinosomes. Altogether, the semi-automated protocol we present provides sufficient accuracy in quantitation of macropinosome numbers, allows quantitation of additional parameters (e.g. macropinosome size and size distribution) that would be hardly possible to assess by manual measurements, and also greatly improves the efficiency at which this assay can be applied to a large scale comparative analysis of high numbers of cells under a wide range of conditions.

## Method detail – quantitation of macropinocytosis

### Dextran-uptake

1. Plate cells on poly-l-lysine (PLL)-coated coverslips in a 24-well dish at 0.05 × 10^6^ cells per well.
  - Quantitation assay on confluent monolayer of the cells is feasible, however using lower cell density (50–75% confluency) allows the cells to spread on the cover slip so that the nascent macropinosomes are easier to detect at the cell periphery and also diminishes artefacts during image segmentation for quantitative analysis.
  - Cells are cultured in 5% CO_2_ incubator at 37 °C and all subsequent treatments are performed at 37 °C using pre-warmed media
2. At least 16 h prior to dextran uptake, exchange the medium for Foetal Calf Serum (FCS)-free medium.
  - Starvation of the cells allows better synchronization of EGF-induced macropinocytosis. When using cells in which prolonged serum starvation induces apoptosis a reduction of serum concentration from 10% to 0.2–0.5% should be used instead.
3. For a negative control, pre-treat the cells with FCS-free DMEM containing 1 mM Amiloride (or DMSO control) for 30 min. Remove medium from cells (including negative controls) and pulse the cells with fluorescently labelled dextran (100 μg/ml) in FCS-free DMEM (37 °C) containing 100 ng/ml EGF in the presence or absence of 1 mM Amiloride (or DMSO control) for 5 min.
  - Using lysine-fixable dextran analogues is essential since carbohydrates are not chemically fixed by formaldehyde solutions (in following steps) and conventional dextrans will thus leak out from fixed cells.
  - To reduce the autofluorescence background in the cells, culturing cells in phenol-red free medium prior and during the dextran uptake is recommended.
  - Concentration of dextran and time of the pulse required for dextran loading into macropinosomes may differ when using different cell types and dextran of high molecular weight.
4. Remove the dextran-containing medium and wash cells briefly with PBS and fix with 4% paraformaldyde in 250 mM Hepes for 30 min.

- When selective labelling of the plasma membrane is required, some lipophilic probes like FM4-64 (Invitrogen) may be applied for about 1–2 min prior cell fixation whereas for example fluorescently coupled wheat germ aglutinin can be also applied post-fixation but prior to cell permeabilization.

### Fluorescence labelling

1. When immunolabelling of intracellular markers or other fluorescent staining is required, the plasma membrane can be permeabilized by saponin (0.1% in PBS) for 10 min.
  - Note that use of non-ionic detergents like Triton-X100 which permeabilize all cellular membranes results in partial leakage of the dextran from macropinosomes even when using commercially available lysine-fixable dextran analogues.
2. Perform the immunolabelling and/or fluorescent staining of the plasma membrane, F-actin or nuclear DNA using standard immunofluorescence protocols.
  - Expression of GFP or utilization of commercially available cell mask stains labelling plasma membrane or stains that allow homogenous fluorescent labelling throughout the whole cell may be required for accurate image segmentation and for definition and measurement of individual cell area especially when macropinosomes to be quantified in cells that exhibit heterogenous morphology and size.
  - For cells with distinctive cortical F-actin, staining with fluorescently labelled phalloidin can be used as an alternative to define edges of individual cells, e.g. within a confluent monolayer.
3. Mount the coverslips on conventional glass slides using a mounting medium containing an anti-fade agent (e.g. Dako fluorescence mounting medium).

### Image acquisition

For imaging protocol using confocal microscopy, the 40× objective (NA = 1.0 or higher) and acquisition at 512 × 512 px resolution (or higher) should be satisfactory to resolve individual macropinosomes as small as 0.3 μm in diameter (and larger) while keeping the field of view large enough to capture many cells per image. To minimize the risk of failing to detect macropinosomes in different confocal planes, the Z-slices throughout the entire height of the cell monolayer should be acquired using constant spacing between slices (0.5 μm).

As an alternative to acquisition of confocal z-stack images for selected field of view, an epifluorescence microscope can also be used to acquire single plane of focus images of the same area. However, this requires an objective with depth of field spanning the entire cell monolayer (5–10 μm) and sufficient camera resolution to discriminate individual macropinosomes using this objective.

Regardless of field of view size and numbers of cells per image, at least 200 cells should be acquired for each sample in triplicate for reliable statistics. To allow quantitative comparison between groups, the entire set of images for all samples must be acquired using the same laser power, gain, offset and other parameters that affect fluorescence signal to noise ratio. Similarly, when using epifluorescence microscope the manual camera exposure and sensitivity must be maintained at constant value.

### Image processing and quantitative analysis using ImageJ

The complete set of images from all samples within an experiment must be processed and analyzed using batch processing or user-defined macro(s) to preserve potential quantitative differences between tested groups of samples. Optional variables used during image processing and segmentation in this section includes (i) rolling ball radius for fluorescence background subtraction and (ii) the threshold limits to generate a binary image. Setting of optimal values for both variables is dependent on signal-to-noise ratio within the original image which may vary between different experiments. Therefore for each experiment, these values should be optimized before proceeding to macro algorithm and batch processing set up for automated segmentation protocol.

1. Import confocal z-stack images to ImageJ (or an alternative image processing application) and split the colour channels to separate red channel (macropinosomes containing dextran–TMR) from green channel (e.g. cells expressing GFP or GFP-tagged construct) and blue channel (nuclei labelled by DAPI).
2. Select the red channel, collapse the z-stack using Maximum intensity projection, convert resulting image to 8-bit grey scale and use background subtraction which utilizes a rolling-ball algorithm to remove smooth continuous background [14].
3. Start image segmentation by setting the threshold and creating a binary image of dextran-positive structures. Optionally, the watershed filter can be applied to separate individual structures in closely packed areas.
  - [Optional] When quantitation restricted to GFP-positive cells is required, first select the green channel from the same z-stack and follow steps 2 and 3 to generate a binary image mask of GFP-positive cells and then use image calculator to superimpose the mask over the macropinosome image generated in step 3. This will exclude all dextran-positive structures beyond the GFP-positive areas.
4. Analyze segmented image of dextran-positive macropinosome by Particle analysis tool with filter size set to an area of 0.2–20.0 μm^2^ which will exclude structures from the analysis, that are below 0.5 μm or over 5.0 μm in diameter.
5. Save measurements of particle counts, total area, average size, integrated density (or additional parameters as required for macropinosome analysis).
6. To calculate cell numbers per image, select the blue channel of the same confocal z-stack and follow steps 2 and 3 to generate segmented binary image of DAPI-stained nuclei.
7. Analyze segmented image by Particle analysis with filter size set to an average nucleus area to exclude other DAPI-positive structures (e.g. mitochondria), save particle counts as a number of nuclei (cells) per image and use the values from step 5 to calculate the average number of macropinosomes (and other measured values) per single cell within an image.

### Validation controls

#### Macropinocytosis increases in response to EGF treatment

**Fig. 1:**
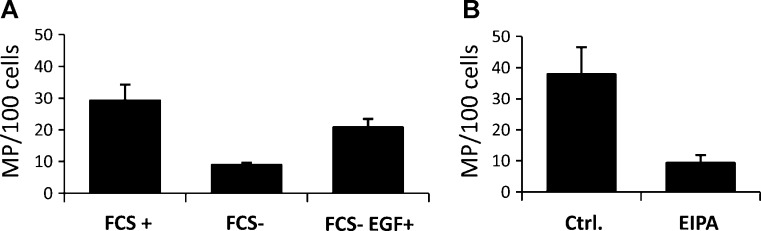
Quantitative macropinocytosis assay and assessment of specificity (A) EGF induces macropinosome formation: Cells were serum-starved for 16 h and incubated with 100 μg/ml dextran–TMR in the presence or absence of 100 ng/mL EGF for 5 min at 37 °C before fixation. MP – macropinosomes; FCS – Foetal Calf Serum and (B) Amiloride inhibits macropinocytosis: Cells were treated with either 1 mM 5-(N-ethyl-N- isopropyl)amiloride (EIPA) or the carrier (0.6% methanol) for 30 min at 37 °C prior the pulse with dextran–TMR (as in A) in the continued absence of the drug or the carrier. The average number of macropinosomes per 100 cells were quantified using the automated image analysis. Error bars represent standard error of the mean (SEM) across three technical replicates of 500 cells per each group.

The EGF has been shown to rapidly stimulate macropinocytosis in A431 human epithelial carcinoma cell line through the activation of PI(3)K [2]. However, these cells express abnormally high levels of EGF receptor what may be an inconvenient characteristic for a model cell line to be used in a quantitative assay. Our laboratory established the HEK293 in the macropinocytosis assay for consistent responsiveness of this cell line to EGF treatment [6]. We found that an overnight serum starvation of HEK293 cells induces 3.3-fold reduction in macropinosome numbers in comparison to cells cultured in the continual presence of serum while treatment of the starved cells with EGF during dextran uptake results in 2-fold increase in macropinosome numbers – almost to the level of cells grown in the presence of serum (Fig. 1A). These results demonstrate that HEK293 cells are sufficiently sensitive to the EGF treatment to be used as a model cell line for quantitative assessment of macropinocytosis.

#### Amiloride is a potent inhibitor of macropinocytosis

Amiloride is a selective Na^+^/H^+^ antiport inhibitor and millimolar concentrations of Amiloride inhibit macropinocytosis but not clathrin-mediated endocytosis [15]. In our protocol we use 1 mM Amiloride or its analogue EIPA, 5-(N-ethyl-N-isopropyl)amiloride. Cells pre-treated with EIPA for 30 min prior to dextran uptake exhibited a 4.5-fold reduction in macropinosome numbers relative to controls (Fig. 1B), indicating that the dextran-positive structures visualized through the assay were sensitive to inhibition of ion exchange or to subsequent lowering of submembranous pH and preventing Rho GTPase signalling and actin remodelling [16].
